# Development of the WHO Environmental Noise Guidelines for the European Region: An Introduction

**DOI:** 10.3390/ijerph15040813

**Published:** 2018-04-20

**Authors:** Dorota Jarosińska, Marie-Ève Héroux, Poonum Wilkhu, James Creswick, Jos Verbeek, Jördis Wothge, Elizabet Paunović

**Affiliations:** 1 World Health Organization (WHO) Regional Office for Europe, European Centre for Environment and Health, Platz der Vereinten Nationen 1, 53113 Bonn, Germany; herouxm@who.int (M.-È.H.); wilkhup@who.int (P.W.); creswickj@who.int (J.C.); wothgej@who.int (J.W.); paunovice@who.int (E.P.); 2 Finnish Institute of Occupational Health, Cochrane Work, Neulaniementie 4, 70701 Kuopio, Finland; Jos.Verbeek@ttl.fi

**Keywords:** noise, WHO environmental noise guidelines, noise abatement, Environmental Noise Directive, health, wellbeing

## Abstract

Following the Parma Declaration on Environment and Health adopted at the Fifth Ministerial Conference (2010), the Ministers and representatives of Member States in the WHO European Region requested the World Health Organization (WHO) to develop updated guidelines on environmental noise, and called upon all stakeholders to reduce children’s exposure to noise, including that from personal electronic devices. The WHO Environmental Noise Guidelines for the European Region will provide evidence-based policy guidance to Member States on protecting human health from noise originating from transportation (road traffic, railway and aircraft), wind turbine noise, and leisure noise in settings where people spend the majority of their time. Compared to previous WHO guidelines on noise, the most significant developments include: consideration of new evidence associating environmental noise exposure with health outcomes, such as annoyance, cardiovascular effects, obesity and metabolic effects (such as diabetes), cognitive impairment, sleep disturbance, hearing impairment and tinnitus, adverse birth outcomes, quality of life, mental health, and wellbeing; inclusion of new noise sources to reflect the current noise environment; and the use of a standardized framework (grading of recommendations, assessment, development, and evaluations: GRADE) to assess evidence and develop recommendations. The recommendations in the guidelines are underpinned by systematic reviews of evidence on several health outcomes related to environmental noise as well as evidence on interventions to reduce noise exposure and/or health outcomes. The overall body of evidence is published in this Special Issue.

## 1. Introduction

Normal sounds become noise when they are unwanted or harmful. Exposure to environmental noise is associated with an increased risk of negative physiological and psychological health outcomes. Although noise is a product of many human activities, widespread exposure to noise from transport (road traffic, railway, and aircraft) is of major concern, affecting the health and wellbeing of many people in Europe. To this effect, environmental noise features among the top environmental hazards to physical and mental health and wellbeing in Europe [1,2].

## 2. Policy Context

The World Health Organization Regional Office for Europe comprises fifty-three Member States covering a vast geographical region from the Atlantic to the Pacific oceans. The European Environment and Health Process and its Ministerial Conferences guide the regional efforts to address the main environmental challenges to human health. The Parma Declaration on Environment and Health, adopted by the Member States of the WHO European Region at the Fifth Ministerial Conference in 2010, made implicit the need to reduce exposure to noise, and called upon the WHO to develop suitable guidelines [3].

At the European Union scale, the Environmental Noise Directive (END) 2002/49/EC offers a common approach to avoiding and preventing exposure to environmental noise, thereby reducing its harmful effects, as well as preserving quiet areas [4]. In implementing this directive, the European Commission is supported by the European Environment Agency (EEA), which gathers the noise exposure data and maintains the Noise Observation and Information Service for Europe (NOISE) [5].

The END (2002/49/EC) is a primary legislative tool for achieving one of the priority objectives of the Seventh Environment Action Programme, of “significantly reducing noise pollution in the EU by 2020 and thereby moving closer to World Health Organization (WHO) recommended levels” [6]. The directive sets out methods for collecting data on noise levels. These END outputs, then, provide a basis for developing measures to reduce noise levels at source. 

## 3. Data on Noise Exposure Levels

The current state of knowledge on noise sources and population exposure in Europe is largely based on data submitted by the member countries of the European Union (EU) on a five-year cycle to the EEA [4,7]. The EEA database covers noise sources specified in the END (such as major roads, major railways, major airports, and urban agglomerations) and number of people exposed to each of the noise sources inside and outside urban areas. Noise levels (L) are calculated and represented in 5-dB interval bands at L_den_ ≥ 55 dB (an average of day, evening, and night) and at night (L_night_) ≥ 50 dB. These long-term average noise exposure indicators are reasonable and common predictors of adverse health effects in a population. However, other noise indicators might be useful to reflect special noise situations. In the case of noisy but short-lived events, like shooting noise or noise emitted by trains, Lmax is often used; for example, Lmax is an indicator of the maximum sound pressure reached during a defined measurement period. Used for setting noise limits, it is also considered in studies to determine certain health effects (e.g., awakening reactions) [8,9].

New scientific evidence, as also illustrated by the systematic reviews published in this Special Issue, shows that health and wellbeing can be affected at lower noise levels than specified by the END [10]; however, as reporting for these lower levels is not required under the END, there is a paucity of data on numbers of the population exposed below 55 dB. Analyses of the available noise exposure data show consistently that the dominant source of noise in Europe is road traffic; the second one is noise from railways, followed closely by aircraft noise. An extension of the mapping of noise exposure to the levels below 55 dB would expand the knowledge base and facilitate the evaluation of progress in preventing adverse health effects. 

Despite substantial progress over the last fifteen years in data mapping and development of noise action plans, there is room for further improvement. For example, in 2013, only 44% of the expected data was delivered in the latest reporting round under the END [11]. In particular, noise exposure data from the eastern part of the Region is lacking, and inconsistencies in quality and quantity of reported data make the discernment of noise exposure patterns difficult. 

The data related to noise sources and exposure information as prescribed by the END, combined with the data on industrial activity, urban areas, land use, and areas protected for the benefit of nature reported by countries to the EEA, were the basis of a first spatial assessment of areas in Europe potentially unaffected by noise pollution caused by human activity [11]. Protection of such areas, largely undisturbed by noise from traffic, industry, or recreational activities is vitally important also from the perspective of human health protection.

## 4. Burden of Disease in Europe 

Noise exposure is associated with a number of adverse health outcomes. Auditory effects of noise include hearing impairment and tinnitus, whilst nonauditory effects refer to cardiovascular and metabolic effects; adverse birth outcomes; poor quality of life, mental health, and wellbeing; annoyance; cognitive impairment; and poor sleep. Sleep disturbance and annoyance, mostly related to road traffic noise, are the most prevalent effects from noise.

The WHO Global Burden of Disease (GBD) measures the burden of disease using the disability-adjusted life-year (DALY) [2], which combines years of life lost due to premature mortality and years of life lost due to time lived in states of less than full health. The DALYs lost due to noise-induced health outcomes in the western part of Europe were estimated to be equivalent to: 903,000 years for sleep disturbance, 654,000 years for annoyance, 61,000 years for ischaemic heart disease, 45,000 years for cognitive impairment of children, and 22,000 years for tinnitus [2]. The burden of disease could not be calculated for the eastern and central part of the WHO European Region due to a lack of reported noise exposure data.

## 5. Why the Urgency?

With projections of rapid urban growth in Europe (80% of the citizens are expected to be living in or near a city by 2020) [12] and increased demand for road, rail, and air transport [13], a simultaneous increase in noise exposure and the associated adverse health effects can be anticipated. Hence, it is pertinent to continue positioning noise mitigation as a cross-cutting theme on the agenda of urban development and transport policies.

Available reported data on long-term average exposure show that 65% of Europeans living in major urban areas are exposed to daytime noise levels greater than 55 dB, and more than 20% to night-time noise levels greater than 50 dB, at which adverse health effects occur frequently [5]. As shown by the Eurostat surveys, noise from neighbours (defined as noise from, e.g.., neighbouring apartments, staircases, or water pipes) and streets (described as noise linked to traffic, business, factories, agricultural activities, clubs, and yard) is also perceived to be a source of annoyance. An estimated 18% of citizens of the 28 member countries of the European Union reported being exposed to noise from neighbours or the streets in their living areas in 2016, a decrease from 24% in 2005 [14,15]. As data on noise created by neighbours is not collected through the noise-exposure mapping process (END 2002/49/EC), such a noise has not been considered in the development of the WHO Environmental Noise Guidelines.

Furthermore, reliance on wind energy has increased in the last years in Europe, with wind farms being an important component of Europe’s shift towards a greener, renewable energy supply. However, the noise from increased installations of wind turbines have resulted in higher public annoyance in the EU [16].

Finally, concerns on the increasing exposure to noise in leisure settings are growing along with operation of personal music devices at unsafe volumes. WHO estimated that young people worldwide could be at risk of hearing loss due to these unsafe listening practices [17]. In the EU, a conservative estimate of users of devices such as personal music players and mobile phones with music functions lies in the range of 50–100 million people [18]. Noting the possible effects of wind turbines and personal devices on health, scientific literature pertaining to these noise sources have been considered in the development of the Noise Guidelines.

## 6. The WHO Guidelines

In 1999 and 2009, WHO published guidelines to protect human health, specifically from community noise and night noise exposure [18,19]. Over the years, there have been a number of key developments and a substantial increase in the number and quality of studies on environmental noise exposure and health outcomes, with newly found associations with annoyance; cardiovascular effects; obesity and metabolic effects (such as diabetes); cognitive impairment; sleep disturbance; hearing impairment and tinnitus; adverse birth outcomes; and quality of life, mental health, and wellbeing. Another development is that whilst earlier studies focused mainly on road traffic and aircraft noise [11], newer studies also include noise from other sources such as railways and wind turbines.

In light of this new evidence, the WHO Environmental Noise Guidelines for the European Region are being developed in accordance with the “WHO Handbook for Guideline Development”, which sets out a clear framework to ensure rigorous adherence to the systematic use of evidence as the basis for developing public health recommendations [20]. Systematic reviews of scientific literature guided by specific key questions using the PICO or PECCOS (population, intervention/exposure, control, confounder, outcome, and study design) structure form the basis of the recommendations in the guidelines. 

The two main questions that frame the guideline recommendations are:

1. In the general population exposed to environmental noise, what is the exposure–response relationship between exposure to environmental noise (reported as various indicators) and the proportion of persons with a validated measure of health outcome, when adjusted for confounders?
2. In the general population exposed to environmental noise, are interventions effective in reducing exposure to and/or health outcomes from environmental noise?

The development of the guidelines involves the collaboration of many groups, including the WHO steering group, the Guideline Development Group (GDG), the External Review Group (ERG) and the Systematic Review Team (SRT). The exact roles and composition of these groups, set out in the WHO Handbook for Guideline Development, are briefly summarized here [20]:

- The prime responsibility of the GDG lies in the development of evidence-based recommendations, based on the outcomes of the systematic reviews of evidence as well as careful consideration of other factors (such as values and preferences, balance of benefits and harms, and resource implications).
- The SRT is comprised of leading experts in the field of environmental noise and health, and their role is to review all relevant literature in the context of the guidelines.
- The ERG is composed of thematic experts as well as stakeholders representing individuals who are likely to be affected by the recommendations and interested parties. They are asked to participate at different stages to comment on clarity and implications for implementation.

Declaration of interests for each member of these groups are collected and managed to prevent bias from conflicts of interest. More information on the types of interests that need to be declared can be found in the handbook.

Seven systematic reviews of evidence were commissioned by WHO to assess the relationship between environmental noise and the following health outcomes: (1) annoyance; (2) cardiovascular and metabolic effects; (3) cognitive impairment; (4) effects on sleep; (5) hearing impairment and tinnitus; (6) adverse birth outcomes; and (7) quality of life, mental health, and wellbeing. An eighth systematic review was commissioned to assess the effectiveness of environmental noise interventions in reducing exposure and associated impacts on health. The reviews separately assess the environmental noise coming from the following sources, for each relevant health outcome: road traffic, railway, aircraft, wind turbines, and leisure. In the context of the WHO environmental noise guidelines, leisure noise was defined as outdoor and indoor exposure during leisure activities (such as discotheques, cafes, festivals, concerts, or personal music devices). Due to the individualized retrieval of evidence for each of the systematic reviews, the timeframes of the included literature varied; an indication of the temporal coverage of the studies included can be found in specific systematic reviews. A detailed description of the methodology used to conduct the systematic evidence reviews, including individual protocols, has been prepared as part of the guidelines development process and will be published on the WHO Regional Office for Europe website.

The key objectives of the systematic evidence reviews were to assess the strength of the association between exposure to environmental noise and incidence or prevalence of adverse health effects, and where possible, to quantify the risk of these health effects with an incremental increase in noise exposure. A detailed description of the methodology used to conduct the systematic evidence reviews can be found in the systematic reviews published in this Special Issue.

WHO has adopted the grading of recommendations, assessment, development, and evaluations (GRADE) approach [20] in order to assess the quality of evidence and develop and report recommendations in the form of guidelines. GRADE is widely acknowledged as an effective method of rating the quality of the evidence and linking evidence to clinical recommendations because this approach facilitates judgments about the certainty in the observed effect estimates and the strength of the recommendations. The limitations to the application of the original GRADE in environmental health have been discussed in the literature [21]. Specifically in the context of the environmental noise guidelines, the GRADE approach was adapted to the observational studies, which are usually the only source of research evidence in this area. The main adaptations made to the GRADE approach for environmental noise are also discussed in the systematic reviews. The upcoming guidelines focus on the WHO European Region and provide policy guidance to its Member States that is compatible with the noise indicators commonly used in the END, namely L_den_ and L_night_.

## 7. Looking Ahead

The evidence summarized and presented in the systematic evidence reviews is the basis for the development of recommendations in the WHO Environmental Noise Guidelines for the European Region. Aimed at decision-makers and technical experts, the new guidelines offer not only scientific, evidence-based rationale for identifying levels, at which environmental noise is related to a significant health impact, but also recommendations for actions to reduce exposure. For all who are involved in health and environmental impact assessment, such as policy makers, advocacy bodies, and researchers, these guidelines make recommendations on noise levels above which we are confident that there are health impacts for some noise sources and provide guidance for quantifying these impacts. Moreover, the guidelines highlight critical data and research gaps to be addressed in future studies. Although developed for the WHO European Region, the guidelines provide a general framework for use by a global audience.

## 8. Conclusions

As policy-makers begin to address rapid urbanization and sustainable economic development, the evidence systematically reviewed as part of the WHO Environmental Noise Guidelines for the European Region offers a useful reference for establishing the links between noise pollution and public health, especially taking into account effects on large populations in urban environments. Governments and communities are encouraged to use the opportunity to champion a multidisciplinary approach to help mainstream noise mitigation in their sustainable development processes.

## References

[1] Hänninen O., Knol A.B., Jantunen M., Lim T.-A., Conrad A., Rappolder M., Carrer P., Fanetti A.-C., Kim R., Buekers J. (2014). Environmental burden of disease in Europe: assessing nine risk factors in six countries. Environ. Health Perspect..

[2] WHO Regional Office for Europe, European Commission Joint Reasearch Centre (2011). Burden of Disease from Environmental Noise-Quantification of Healthy Life Years Lost in Europe.

[3] WHO Regional Office for Europe Parma Declaration on Environment and Health. Proceedings of the Fifth Ministerial Conference on Environment and Health.

[4] European Commission (2002). Directive 2002/49/EC of the European Parliament and of the Council of 25 June 2002 Relating to the Assessment and Management of Environmental Noise.

[5] (2018). European Environment Agency. https://www.eea.europa.eu/themes/human/noise/noise-story-map.

[6] European Commission (2013). Environment Action Programme to 2020 “Living Well, within the Limits of Our Planet”.

[7] European Environment Agency (2012). EEA Technical Report No 9/2012 Electronic Noise Data Reporting Mechanism.

[8] Basner M., Isermann U., Samel A. (2006). Aircraft noise effects on sleep: Application of the results of a large polysomnographic field study. J. Acoust. Soc. Am..

[9] Elmenhorst E.M., Pennig S., Rolny V., Quehl J., Mueller U., Maaß H., Basner M. (2012). Examining nocturnal railway noise and aircraft noise in the field: sleep, psychomotor performance, and annoyance. Sci. Total Environ..

[10] Guski R., Schreckenberg D., Gemeinnützige Umwelthaus gGmbH (Hg.) (2015). Gesamtbetrachtung des Forschungsprojekts. NORAH. Verkehrslärmwirkungen im Flughafenumfeld (Bd. 7).

[11] Blanes N., Fons J., Houthuijs D., Swart W., de la Maza M.S., Ramos M.J., Castell N., van Kempen E. (2017). Noise in Europe 2017: Updated Assessment.

[12] Eurostat (2017). Urban Europe—Statistics on Cities, Towns and Suburbs.

[13] European Commission (2016). EU Reference Scenario 2016—Energy, Transport and GHG Emissions: Trends to 2050.

[14] Eurostat Noise from Neighbours or from the Street—EU-SILC Survey. http://ec.europa.eu/eurostat/en/web/products-datasets/-/ILC_MDDW01.

[15] Eurostat (2015). Quality of Life: Facts and Views.

[16] Van Renterghem T., Bockstael A., De Weirt V., Botteldooren D. (2013). Annoyance, detection and recognition of wind turbine noise. Sci. Total Environ..

[17] World Health Organization (2015). Hearing Loss due to Recreational Exposure to Loud Sounds: A Review.

[18] Scientific Committee on Emerging and Newly Identified Health Risks (SCENIHR) (2008). Potential Health Risks of Exposure to Noise from Personal Music Players and Mobile Phones Including a Music Playing Function.

[19] Berglund B., Lindvall T., Schwela D.H., World Health Organization (1999). Guidelines for Community Noise.

[20] World Health Organization (2014). WHO Handbook for Guideline Development.

[21] Morgan R.L., Thayer K.A., Bero L., Bruce N., Falck-Ytter Y., Ghersi D., Guyatt G., Hooijmans C., Langendam M., Mandrioli D. (2016). GRADE: Assessing the quality of evidence in environmental and occupational health. Environ. Int..

